# A Review with Updated Perspectives on Nutritional and Therapeutic Benefits of Apricot and the Industrial Application of Its Underutilized Parts

**DOI:** 10.3390/molecules27155016

**Published:** 2022-08-07

**Authors:** Maryam Haroon Al-Soufi, Hussah Abdullah Alshwyeh, Haifa Alqahtani, Safa Khalil Al-Zuwaid, Fatimah Othman Al-Ahmed, Fatima Taher Al-Abdulaziz, Daniya Raed, Khaoula Hellal, Nurul Hidayah Mohd Nani, Siti Norliyana Zubaidi, Nurul Syahidah Mio Asni, Hamizah Shahirah Hamezah, Nurkhalida Kamal, Hessah Al-Muzafar, Ahmed Mediani

**Affiliations:** 1 Department of Biology, College of Science, Imam Abdulrahman Bin Faisal University, Dammam 34212, Saudi Arabia; 2 Basic & Applied Scientific Research Centre, Imam Abdulrahman Bin Faisal University, Dammam 31441, Saudi Arabia; 3 Department of Chemistry, Muğla Sıtkı Koçman University, Muğla 48121, Turkey; 4 Institute of Systems Biology, Universiti Kebangsaan Malaysia (UKM), Bangi 43600, Malaysia; 5 Department of Chemistry, College of Science, Imam Abdulrahman Bin Faisal University, Dammam 34212, Saudi Arabia

**Keywords:** apricot, bioactive compounds, health benefit, industrial application

## Abstract

Fruits maintain the image as the richest sources of vitamins. Focusing on apricots, utilization of apricot species for many applications is possible due to its various benefits. Many research studies demonstrated different perspectives of apricot, especially in medical used as it can act as antioxidant, anti-inflammatory, and antimicrobial agents. Moreover, in the industrial sectors, apricots can be used in the production of biofuels and batteries. All components of the apricot fruit, including seeds and kernels have been found to possess significant interest. This review is to breach the knowledge gap regarding the key nutrients and chemicals of apricot fruit, contributing to its health-promoting properties to emphasize the noble importance of this fruit in the diet and in the management of several diseases. We also cover the application of apricots in the industry that could be developed as a promising and sustainable source.

## 1. Introduction

Due to the myriad health benefits of fruits, their consumptions have been encouraged. There are also numerous epidemiological studies suggest that the intake of vegetables, and fruits as well as their products may reduce the risk of several chronic diseases and delay the ageing process [1]. The propagation of apricot can be carried out from seeding in a small area to a large area and it can be also vegetatively propagated. It is one of the most commercially important fruits, which occupies the third position after plum and peach due to its nutritional value and medicinal properties that make it as “golden fruit” [2]. Its name derived from Latin word “praecocia” meaning “early ripening fruit” [3]. Apricot fruit encompasses various type of species that belong to Rosaceae family in the genus of *Prunus*, known as stone fruit [4]. Universally, an apricot tree is known as *P. armeniaca* species. Other species, including *P. mume, P. brigantina, P. zhengheensis, P. mandshurica,* and *P. sibirica* are also called as apricot because they are hierarchically related and have identical fruit [5]. Apricot fruit is usually consumed raw or processed into juice, beverages, lyophilized products, jelly, and jam because of its delicacy and health benefits.

Apricot phytochemical composition is influenced by a number of factors, including variety, maturity, environmental conditions, cultivation practises, soil structure, and applied fertilizers [6]. Apricot fruits are rich in phenolic and carotenoid compounds, which are involved in many functions, including taste and colour [7]. Many phenolics were identified in the apricot (i.e., chlorogenic, gallic, ferulic, salicylic, and p-coumaric acids) and flavonol (i.e., glycosides, rutin, quercetin, and vanillin) [2,8]. Higher concentrations of phenolic and carotenoid compounds were found to be present in fruit peel or flesh and the total phenolic composition ranged from 50.00 to 563.00 mg Gallic Acid Equivalents (GAE)/100 g on fresh weight basis [3]. The other identified compounds include catechin, epicatechin, neochlorogenic acid, chlorogenic acid, cyanidin-3-glucoside, lutein, zeaxanthin, violaxanthin, and β-cryptoxanthin as well as α-, β- and γ-carotene [9]. 

Apricots are mostly grown throughout Europe and the Mediterranean, with the majority of available data coming from cultivars grown in these temperate to subtropical climate zones [8]. Apricot fruits are normally accessible for consumption once a year due to weather conditions. Cultivation in this region is difficult due to severe weather circumstances, as well as this fruit tree’s intrinsic limitations to environmental adaptation, which limits productivity. Apricots are climacteric fruits that can ripen on or off the tree. The time of harvest is thus largely determined by the intended market and/or purpose of the fruit, with produce tended for distant markets or extended storage for early harvested fruits than those for local markets, with the latter group considered to have better eating quality despite its shorter shelf life. The effect of these techniques, particularly on fruit ripeness during harvest, has gotten some attention. Dragovic-Uzelac et al. [10] reported that the phenolic compounds in these stone fruits are prevalent during the starting and early phases of growth, but they may decrease with maturity; contradictory findings have been reported [9]. In addition, Iordanescu et al. [11] reported that protein and moisture of seven apricot cultivars were studied during three fruit ripening stages reduced with maturity, while lipids, carbohydrates, and ash content are increased during ripening. Ripening has also been linked to an increase in carotenoid concentration. The impact of these parameters on bioactive components and nutritional content of apricots should be studied in research on resultant variations. However, thorough scrutiny of literature apricot was not backed adequately. The focus of this research is to summarize the most recent research in this field with a focus on therapeutic action, bioavailability, and phytochemicals as well as industrial applications. 

The apricot has a wide range of potential health advantages. As part of a healthy diet, regular eating of fruits and vegetables, especially apricot, may help avoid chronic disease and maintain better health. In order to further understand the mechanism underlying the apricot’s capacity to lower risk of various disease, researchers should continue to investigate the interactions between the numerous phytochemicals found in apricots. Therefore, this review is to breach the knowledge gap regarding the key bioactive compounds of apricot fruit and its health-promoting properties to emphasize the noble importance of this fruit in the diet and in the management of several diseases. In addition, it is advocated that apricot and plum seed oils replace other vegetable oils. Thus, we sought to assess the potential valorisation procedures for its bio wastes to optimise their advantages and investigate the use of apricots in medicine as well as the potential applications of their waste in the food and health food businesses.

## 2. Apricot Production

**Figure 1:**
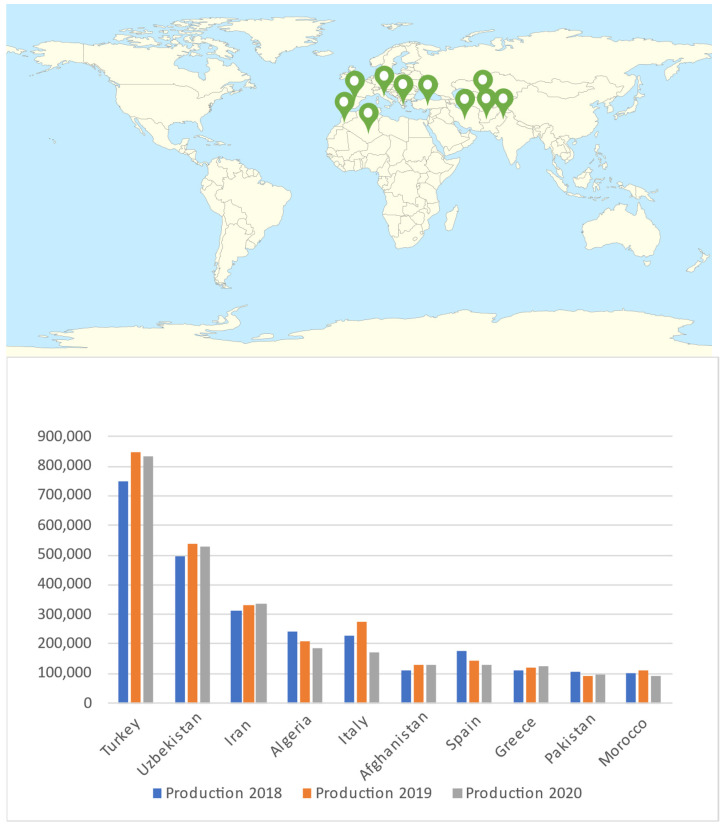
Production of apricot for the top 10 Countries in the years 2018, 2019 and 2020.

**Figure 2:**
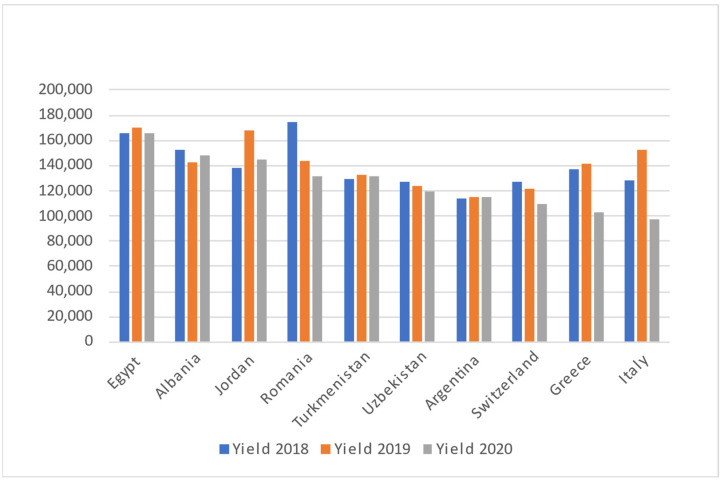
Yield of apricot for the top 10 Countries in the years 2018, 2019 and 2020.

**Figure 3:**
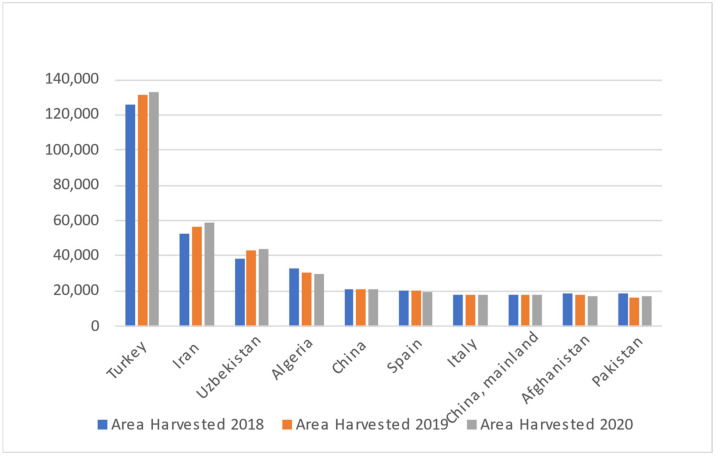
Area harvested of apricot for the top 10 Countries in the years 2018, 2019 and 2020.

Apricot germplasm is classified into four major ecogeographical groupings with regional subgroups (Central Asian, Irano-Caucasian, European, and Dzhungar-Zailij) based on morphological and physiological characteristics. North and East Chinese groups were recently included in the classification [12]. Since apricot seed was first brought to Europe, Middle Asia, and smaller parts of Asia via "the Silk Road", its natural habitats range from Turkistan to the western parts of China. According to Food and Agriculture Organization (FAO), Turkey and Uzbezkistan are the biggest producers of apricots in the world, with 833,398 and 529,109 tons in 2020, respectively. Iran, Algeria, Italy, Afghanistan, Spain, Greece, Pakistan, and Morocco are the subsequent biggest producers. Based on Figure 1, it shows the apricot production by top 10 countries and continent from three years: 2018, 2019, 2020. The top countries of Apricot production are Turkey, Uzbekistan, Iran, Algeria, Italy, Afghanistan, Spain, Greece, Pakistan, and Morocco. From Figure 1, Turkey shows the highest production of apricot for all the years, meanwhile Morocco shows the least production among these countries throughout those three years. The production of Apricot may be affected by their geographical factors/locations and its climate changes. In year 2020, the apricot production showed gradually decrease, which might be because of COVID-19 pandemic. Yield of apricot identifies as the output as an indicator of productivity, efficiency, and quality production process. The Figure 2 shows the yield is significantly consistent for almost of the countries throughout the three following years. In year 2018 and year 2019, the yield of apricot production by most countries showed progress. Meanwhile, in year 2020, all the 10 countries have shown decrease result due to the COVID-19 pandemic that hit the whole world. For China, Spain, Italy, China Mainland, Afghanistan, and Pakistan, have constantly shown they owned the lowest area harvested of apricot production in the year 2018, 2019 and 2020. Based on Figure 3, only Turkey has highest area harvested among others. China, Spain, Italy, China Mainland, Afghanistan, Pakistan showed no changes in all studied years.

## 3. Physical Properties and Taste of Apricot Kernels

Apricots have distinctive physical properties and taste that help to differentiate between species. A study has been carried out to evaluate the physical properties between 11 types of apricots since these characteristics is crucial to determine the design of harvesting and post-harvesting equipment, transportation, cleaning, separation, size, packaging, and processing into various culinary products. The physical properties that are being evaluated are geometric mean diameter (GMD), thickness (T), surface area, width, length, fruit weight, sphericity, true density and pit ratio [13]. Geometric mean diameter will indicate the size of the fruit meanwhile pit ratio means edible bulk of the fruits. Cuminis Haley and Harcot has the highest pit ratio, while the highest geometric mean diameter possessed by Harcot and Cuban. Apricots with character of high ratio pit and large geometric mean diameter are best suited for fresh consumption and the creation of value-added goods, such as jam, juice, and jellies due to their greater size and higher pit ratio [14]. On the other hand, fruit with low weight is suitable for drying and Khante has the least weight [15]. 

**Figure 4:**
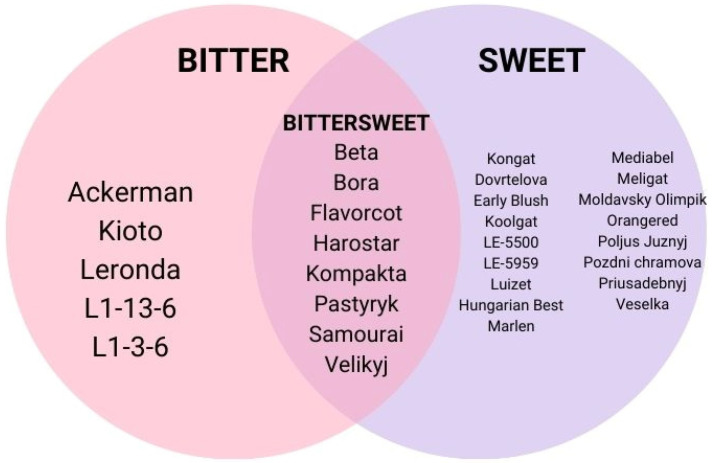
Variation of apricots cultivars based on taste [17].

Sweet kernel taste is the common taste and the most desirable quality in apricots. Other than this taste, some kernels taste bitter, which usually grown in Europe. Apricot seed bitterness is caused by the presence of the cyanogenic glucoside amygdaline after breakdown by chewing. Amygdaline is enzymatically converted into benzylaldehyde and cyanide. Hence, the bitterness level of kernels is determined by the amount of amygdaline. Sweet kernel is valuable in which the seeds can be utilized as almonds [16]. Studies have shown the difference between species depending on those properties (Figure 4) [17]. Figure 4 indicates that the most abundant taste is sweet. However, bittersweet and bitter taste are of approximately equal occurrence in other species, whereas weight of kernels is ranged from 0.7 to 1.7 g [17]. 

## 4. Nutritional Values of Apricots 

Apricot has been phytochemically studied because of its nutritional value (sugars, organic acids, and minerals) and nutraceutical traits (total phenolic, total flavonoids, total carotenoid, and antioxidant activity) and because it is rich in bioactive phytochemicals that contribute to medicinal health benefits [18]. It also characterised by its morphological aspects (fruit weight, flesh/seed ratio, fruit firmness, and colour index). Apricot fruits are a rich source of fibres that prevents constipation and stimulates normal gastric motility [3]. Soluble fibre keeps blood sugar level stable by lowering blood cholesterol, and helps in reducing body weight [19,20,21,22,23,24].

Apricot fruits are a good source of sugars, including sucrose, glucose, fructose, and maltose. Vitamins C, E, B6, and A, as well as minerals and trace elements, are mostly represented by potassium, phosphorus, calcium, and magnesium with minor amounts of iron, sodium, and zinc. The most major organic acids found in apricots are malic acid (500–900 mg/100 g), citric acid (30–50 mg/100 g), and tartaric acid [25]. The kernel contains 14.1 to 45.3% protein, with the primary proteins being albumin, globulin, glutelin, and prolamin with 84.7, 7.65, 3.54, and 1.17%, respectively (Table 1) [17].

Apricots can be consumed fresh or as dried fruit, both of which contain nutritional values. Nutritional characteristics are highly dependent on the apricot cultivars, cultivation systems, and storage conditions [20]. Apart from their nutritional value, apricots contain phenolic compounds, such as chlorogenic acid, catechin, epicatechin, and rutin [17]. The importance of these compounds is to increase the antioxidant activity and are required in food because of the possible relationship between their content and the lower incidence rates of cancer and cardiovascular diseases [21].

This review paper covers fundamental chemical features, including dry matter, soluble sugar, pectins, organic acids, titratable acidity, ash, fibre, etc. Apricots are climacteric fruits that mature quickly after harvesting, limiting their storage period significantly. Thus, different preservation procedures, such as canning, freezing, drying or controlled atmosphere packaging are widely applied to preserve the fruits [3,22]. The most frequent method for preserving apricots and extending their availability is drying [23]. The technique decreases the moisture content of apricots to the point that they can be safely stored for an extended period but on the other hand, the relative concentration of nutrients including vitamin A, vitamin E, potassium and iron is increased [24]. However, several studies have discovered that the antioxidant content of fresh fruits can be altered by the processing method, which can degrade the benefits. Therefore, fresh apricots were dried to see if the effects of two drying procedures, oven-drying (OD) and traditional sun-drying (TSD) [22], had any effect on apricot carotenoids and phenolic compounds (*Prunus armeniaca* L.).

Certain studies had been carried out to evaluate the nutritional value between different species of apricot and it showed that each fruits contain different compositional properties. The study involved Malatya apricots from Turkey region that cover the property of total phenolics, β-carotene, organic acids, total carotenoids, sugars and minerals [25]. The data of nutritional and chemical compositions of several apricot varieties is summarized in Table 2 and 3. It has been clarified that phenolic compound groups such as flavonols, anthocyanins, procyanidins and hydroxycinnamic acid can be found within the various apricot varietals [26]. Hacıkız composes the highest level of total phenolics compared to other Malatya apricot. Tokaloğlu and Bursa varieties contain in range of 4233.7 to 8180.5 mg of GAE/100 g dry weight. Furthermore, total phenolic compounds ranged from 326 to 1600 mg/100 g fresh weight in Spanish apricot varietals [27]. Furthermore, total carotenoids in various type of apricot is significantly different which may include β-cryptoxanthin, lycopene, lutein, β-carotene and γ-carotene [28]. Hasanbey and Kabaası are the Malatya apricot cultivars with the greatest total carotenoid concentrations. Meanwhile, total carotenoid in Spanish apricot is in between 1.36 to 38.52 mg/100 g [25]. On the other hand, the amount of β-Carotene in total carotenoids ranged from 39% to 65%. Studies showed that Alyanak contains the highest content among Malatya apricot varieties [26]. β-Carotene is included under vitamin A and this indicate that apricot can become a good source of vitamin A to human being as this kind of nutrient cannot be synthesized by our body.

The amount of organic acid is significantly different in Malatya apricots in which Iğdır and Bursa varieties has the highest amount of citric acid meanwhile malic acid content is high in Cataloğlu and Hasanbey [25]. Furthermore, among the Malatya apricot types studied, the Hasanbey variety exhibited the highest vitamin C content. In certain study, it reveals that Malatya apricot exhibit more vitamin C compared to other apricots varieties [29]. A study showed that the main sugar in apricot are glucose, sucrose and fructose in which all type of sugars is significantly different in various apricots [30]. Based on study, sucrose is found as predominant sugar in Malatya apricot. Hacıhaliloğlu and Tokaloğlu varieties contain the most amount of sucrose and the highest level of fructose can be found in Cöloğlu and Cataloğlu varieties (Table 3). The higher sugar content and lower organic acid concentrations found in Malatya apricots provide the ideal sweetness and flavour [25]. Moreover, macro and micro elements of mineral also can be found in apricot varieties in which potassium is the most mineral that can be found and sodium is the least one for macro elements. Study has discovered that apricot is one the fruits with good source of minerals hence, it can achieve the demand of mineral needed by average adult [31]. 

Organic acids (OA) and sugars contribute greatly to fruit sensory consistency by adding a pleasant taste and scent [18]. The OA are involved in a variety of bio-logical processes. They inhibit the growth of bacteria, which aids in the preservation of fruits. Furthermore, OA can permeate across cell membranes and breakdown into subsequent ions and protons, hastening the onset of metabolic problems in cells caused by increasing intercellular acidity [32]. Furthermore, these OA can help to stabilise water-soluble vitamins B and C, as well as stimulate appetite and digestion, as well as absorption of minerals including potassium, copper, zinc, iron, and calcium. Additionally, due to their ability to chelate metals, OA may act as antioxidants, earning them the designation of synergistic or preventive. Antioxidants play a major role on the protection of many diseases including cardiovascular diseases, cancer, and inflammation [33].

To extract antioxidants from apricot, dried apricots were rehydrated at room temperature for 24 h [23,34]. To replicate the water content of fresh fruit, samples were rehydrated with the exact amount of water lost during the drying process. The significant loss of water during the drying process resulted in a concentration of the various apricot components, indicating the increase in ash content and acidity [22]. Prior to analysis, samples were homogenised for 2 min. The moisture (MC) and dry matter (DM) content of the obtained purees were determined. They were quantified in a vacuum oven set to 105 °C for 3 h (for fresh, dried, and rehydrated fruit), and the pH was determined (using a digital pH metre) according the previous studies methods [35,36] Then, titration with 0.1 N sodium hydroxide to an endpoint was used to determine the acidity (pH 8.3) and also the ash content was determined after five hours at 550 °C in a muffle furnace (%).

## 5. Biological Activities of Apricot

### 5.1. Antioxidants 

**Figure 5:**
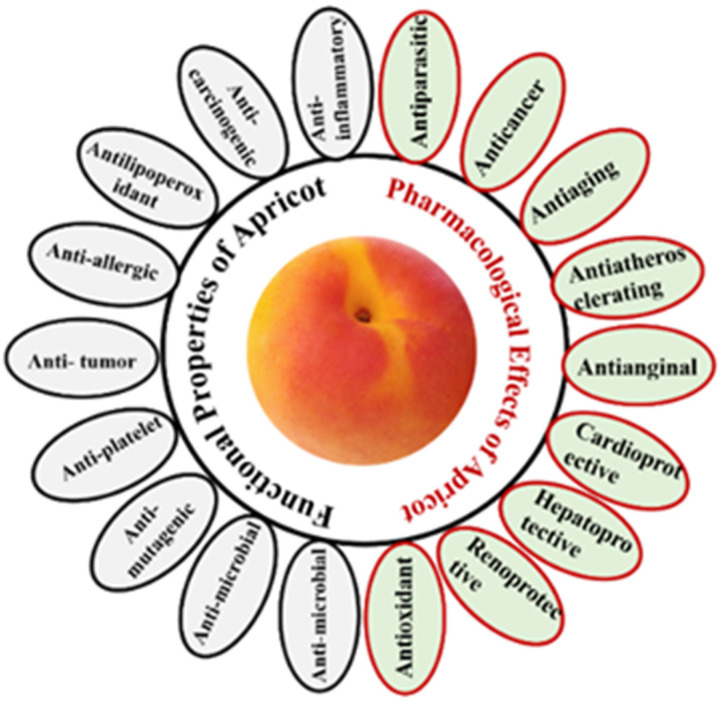
Functional Properties and Pharmacological Effects of Apricot [3,24].

Cardiovascular diseases are the most common cause of death worldwide. Antioxidants components in apricots known for their effectivity in combating coronary heart diseases. Phenolic components in apricots combat the oxidation of low-density lipoprotein (LDL) and thus stimulate the antioxidative status of the body [19]. Figure 5 presents several functional properties and pharmacological effects of apricot as have been reported [3,24]. Apricot vitamins have a high capacity for scavenging free radicals and are found in all apricot species. Apricots’ antioxidant activity benefits a variety of medical conditions by enhancing the body’s defence mechanisms against free radicals and reducing the oxidative effect of free radicals [33,37]. Numerous studies reported shows the great potential of apricot for fighting off free radicals, and thus it considered as a functional food [19].

These conditions include chronic gastritis, oxidative intestinal damage, hepatic steatosis, atherosclerosis, coronary heart disease, and tumour formation. Additionally, apricots contain soluble dietary fibres that contribute to LDL cholesterol reduction [19], resulting in improved heart and liver health. Numerous apricot species are high in vitamins, which aid in the antioxidant pathways that help prevent a variety of illnesses and diseases including degenerative diseases, i.e., cancer, cardiovascular and haemostasis [3]. 

Flavonoids also have an effective function for fighting off free radicals. Being used as antioxidants for the benefit of living organisms. Measuring total flavonoids in apricot kernels showed promising results [21,38]. Several valuable effects of flavonoids in diseases including cardiovascular, some forms of cancer, Parkinson’s and Alzheimer’s diseases have been reported [6]. Carotenoids act as antioxidants and play a crucial role by fighting the reactive oxygen species that cause oxidative damage to living cells [19]. The total flavonoid content (TFC) of various apricot cultivars were tested and the cultivar Bora possessed the highest value of TFC with 153 mg CE/100 g dry weight while no flavonoid content was detected in the Congat and Velikyj cultivars [17]. 

DPPH radical scavenging activity of apricot varieties are significantly different. Study on 14 different types of apricot including Halma, Rakchekarpo, Khante, Shakanda, Viva Gold, CITH-1, CITH-2, Newcastle, Turkey, Nugget, Venatchaa, Shakarpara, Rakausilk, and Sterling showed that Rakausilk variety possesses high antioxidant activity followed by Viva Gold and Rkchekarpo. Furthermore, CITH-1 appears to have low activity of radical scavenging meanwhile Nugget and Shakarpa variety has no significant difference [39]. 

### 5.2. Anti-Inflammatory Activity

Apricot seeds and kernels play a major part as an anti-inflammatory. Apricots contain many significant compounds that reduce inflammation in diseased tissues of animals and humans. A study was made in 2018 for the anti-inflammatory effect of apricot 70% and 99.9% ethanolic extracts of apricot seeds in formalin induced paw edema in rats indicated significant reduction in the inflamed tissues (Figure 5) [40]. Studies support the presence and activity of fatty acids in apricot kernel oil with anti-inflammatory properties [21].

### 5.3. Anticancer Effect of Apricot

Cancer is currently the most prevalent kind of degenerative illness; it is a group of diseases characterized by unrestricted cellular growth and the spread of aberrant cells to other sections of the body. The cancer hallmarks are biological characteristics that evolve during tumour development [19]. These include insensitivity to anti-proliferative drugs, apoptosis avoidance, infinite reproduction, inducing angiogenesis, and beginning invasion of local tissues and metastasis to distant regions [41,42,43,44,45].

The use of complementary and alternative medicine is becoming more popular. CAM has evolved gradually over the previous 15 years and has undeniably gained therapeutic, commercial, and sociological significance. Dietary supplements are the most popular CAM treatments. In apricot kernels the main component to have major effects on cancer is Amygdalin. It was used as an anticancer agent since the 1800s [3]. Amygdalin is a naturally occurring anti-cancer substance that is classified as a β-cyanogenic glycoside found in abundance in Rosaceae family members such as almonds, apricots, apples, and peaches. It is thought that amygdalin’s anticancer properties are attributable mostly to its active metabolite, hydrocyanic acid [46]. Amygdalin is being examined extensively for its potency in cancer cells. Human prostate cancer cells (DU145 and LNCap) exhibited multiple apoptotic markers after amygdalin treatment, including increased Bax generation and decreased Bcl-2 expression, leading to caspase-3 activation. 

Another investigation found that amygdalin alone had suppressive properties and its components (activated with β-d-glucosidase) on proliferation rate of human hepatocellular carcinoma cells [47], i.e., HepG2. Its activated components had a higher apoptotic efficiency than amygdalin. Apoptosis’ specific mechanism is still being researched. This combo method may allow for the use of a low-dose medicine. The extract of Japanese apricots or *Prunus mume*, contained MK615, which inhibits the growth of cancer by dual inhibition of Aurora A and B kinases [48]. MK615 exerts anti-neoplastic effects on several known cancer growths including gastric, breast, and colon cancers and hepatocellular carcinoma. It is also known to induce apoptosis, programmed cell death, autophagy and suppresses A kinase production in cancer growth.

As a result of the anti-inflammatory and antioxidative actions of MK615, the hepatoprotective effects of MK615 was investigated [49]. Furthermore, the benefits of Misatol ME, an MK615-containing beverage approved as a health food product in Japan, were studied in patients with liver illnesses such as hepatitis C, chronic inflammation of the liver, and fatty liver disease, which is linked to oxidative stress.

An investigation had been carried out on 19 apricot varieties from northern Pakistan and Balaani variety has the highest amount of amygdalin meanwhile Staa Chuli contains the lowest content of amygdalin. Amygdalin content commonly appears high in less sweet cultivars [50]. 

### 5.4. Biological Activity of Apricot Leaves

Some of the leaves have been reported to be utilised as traditional treatments by various indigenous populations for the management of rheumatism, asthma, colds, diabetes and urinary tract inflammation. Certain studies stated that the leaves possessed property of antimicrobial, antidiabetic, anticancer, anti-inflammatory, antioxidant, and hypo-cholesterol activities. 

## 6. Medicinal Properties of Japanese Apricot (*Prunus mume*)

The Japanese apricot (*Prunus mume*) enjoys great popularity in Japan. In addition, known as Chinese plum, belongs to Prunes Genus as does the peach, cherries, plum, almonds [51]. It is native to Japan and Korea and is widely planted throughout China. *Prunus mume* is a traditional medicinal herb and health food that has been utilised in Asian countries for over 2000 years because of its health benefits. *P. mume*’s fruit is used to make pickled plums, plum sauce, plum juice, and plum liquor, which can be eaten as a snack, condiment, or beverage. [52]. It is also economically valuable, as it can be processed into juice and wine. These products have been known to possess various medicinal benefits and have been frequently prescribed as a traditional folk remedy. It has been reported to possess such beneficial biological activities. This Japanese apricot is known to have anti-microbial properties and it is the only one well established for various medicinal purposes.

### 6.1. Phytochemical Constituents

The total chemical compounds have been identified in the *P. mume* was 192. Flower buds, fruit, wood, petal, and seed are all high in phenolic components. Phenylpropanoid sucrose esters, hydroxycinnamoylquinic acid derivatives, flavonoids, and other phenolics can all be subdivided. In general, organic acid, steroids, terpenes, lignans, furfurals, benzyl glycosides, cyanogenic glycosides, and alkaloids are among the medicinal chemical components found in the fruit [52].

### 6.2. Medicinal Usage of P. mume Phenolic Compound

#### 6.2.1. Antidiabetic

Diabetes Mellitus (DM) is one of the most common metabolic illnesses in the world. Hyperglycemia is characterized by abnormal insulin production and insulin resistance. Obesity, which is generally accompanied with hyperglycemia and insulin resistance, is one of the most significant risk factors for DM. An aqueous extract of *P. mume* fruit combined with Lithospermum erythrorhizon root increased insulin sensitivity and reduced visceral obesity in rats fed a high-fat diet (HFD). Furthermore, a 70% ethanol extract of *P. mume* fruit and leaves has been shown to increase glucose uptake in C2C12 myotubes, improve fasting glucose levels and glucose intolerance, reduce body weight, and lower blood glucose levels in a dose-dependent manner; this activity is attributed to the phenolic compound. In addition, phenolic extracts of *P. mume* inhibited small intestine disaccharide activity and raised postprandial blood glucose levels in rats. The phenolic compounds extracted from the flower buds of *P. mume* show potential to prevent cataracts [52]. The phenolic extracts from apricot have cardioprotective activity in rats [3]. 

#### 6.2.2. Helicobacter Pylori-Related Chronic Gastritis

Helicobacter pylori infection is linked to chronic gastritis. In vitro and in vivo, *P. mume* extract shows direct bactericidal activity against *Helicobacter pylori* [52]. *H. pylori* motility is required for migration and colonization. The active component syringaresinol isolated from *P. mume* inhibited *H. pylori* motility by 90% at a concentration of 500 mg/mL, with an IC_50_ value of 50 mg/mL, lowering its colonization in the stomach mucosa [53]. A study conducted on non-elderly individuals (65 years) with high-intake (X3 JA daily) significantly reduced *H. pylori* load, mononuclear, and neutrophil infiltration in the gastric mucosa, as well as the preventive effect of dried or pickled *P. mume* on chronic atrophic gastritis (CAG) by inhibiting the infection and reducing active mucosal inflammation. [54].

#### 6.2.3. Antimicrobial and Antiviral Activity

Studies have shown that apricots possess a broad array of antibacterial activities. Consequently, two independent research groups have found that apricots prevent common periodontal bacterial infection, such as *Aggregatibacter actinomycetemcomitans* and *Porphyromonas gingivalis* as well as inhibit the growth of bacteria in the oral cavity [52]. Citric acid, chlorogenic acid, isoquercitrin asparagine, and epicatechin were isolated from ethanol extracts of *P. mume* [55]. These compounds exhibited antibiotic activities against five food-borne bacteria: *Listeria monocytogenes, Salmonella enterica, Bacillus cereus, Staphylococcus aureus*, and *Escherichia coli*. Compared to other compounds citric acid had higher-ranking activities against all five bacteria, chlorogenic acid inhibited four of the five bacteria, epicatechin inhibited *Salmonella enterica* and *Listeria monocytogenes*, while asparagine and isoquercitrin hindered *Listeria monocytogenes* only [12].

Moreover, *P. mume* with *Schizandra chinenis* H-20 show antibacterial activity against *B. subtilis, S. aureus, E. coli* KCCM 11591, and *Pseudomonas aeruginosa* KCTC 1750. The DNA of *S. chinenis* H-20 exhibits inhibition of *B. subtilis, S. aureus, and E. coli* KCCM 11591 at the beginning of cultivation and even after 5 h of initiating cultivation [53]. When *P. mume*, *Schizandrae* Fructus, and Coptidis Rhizoma combined, this led to the inhibition of some *Escherichia coli* and *Salmonella* strains. In addition, *P. mume* inhibits the release of toxins from some strains of *E. coli*. Furthermore, umesu phenolics obtained from *P. mume* revealed inhibition of the multiplication of herpes simplex virus (HSV) and might inhibit superficial HSV infections [52].

## 7. Industrial Utilization of Apricot Seed Waste and Kernels

### 7.1. Applications in Food Industry

Agricultural waste is generally underutilized, and their accumulation in the environment could lead to severe pollution if treated improperly. Apricot (*Prunus armeniaca*) is one of the world’s most extensively cultivated crops due to its nutritional value. One of the options to be explored is apricot stone, seed/kernel and shell. Food sources of protein, fat, and fibre can be found in apricot kernels. All parts of apricot are renowned for its numerous industrial applications, including food, cosmetic, and pharmacy as well as the thermal energy storage. Most critically, it appears that applications for apricot seeds and kernels in the food industries have been not well studied. The apricot kernel is very useful in the food industry for making low-fat biscuits, cakes, pastries, and antimicrobial films. Apricot kernel flour has been claimed to be a good source of minerals, protein, bioactive substances, and fibre, and it has mostly been utilized for product protection in the bakery business. Apricot kernels are primarily utilized in the manufacturing of oils and benzaldehyde; nevertheless, the kernels are also used to baked items, either whole or crushed, and eaten as an appetizer [56]. The kernel powder was defatted and employed as a protein source in yoghurt and ice cream in the proportions of 10–40% and 10–50%, respectively [57]. Similarly, the addition of apricot kernel powder to yoghurt reduced the lactic acid bacteria count, pH, and acetaldehyde value [58]. It was determined that skim milk could be substituted in yoghurt up to a 20% level [59]. Apricot kernel powder was found to improve the quality of stirred yoghurt in a study. Cow milk was replaced with one percent apricot kernel powder. The inclusion of apricot kernel powder enhanced the titratable acidity, ash content, total solids, and protein content [60]. To maintain the quality of the fortified products that are customised for consumers, further studies should also take optimization and the ideal ratio of each waste into account. We can suggest using apricot seeds in the production of innovative juices rich in bioactive peptides, baked goods, veggie-meat-based products, and industrial fermentation to manufacture various food additives such as enzymes, proteins, and flavours.

Apricot kernels are a by-product of the food canning industry in vast numbers. The kernels are viewed as an unconventional source of oil due to their potential and are also rich in unsaturated fatty acids, vitamin E active compounds, triterpenoids, carotenoids, phytosterols, and polyphenols. The strong demand for and consumption of apricots are discarded yearly at processing plants [55] has helped alleviate the environmental problem caused by residue disposal or incineration. Additionally, food sustainability is a critical objective that must be achieved to avoid wasting natural resources and mitigate future effects on climate change and global economic shifts. Utilising apricot oils from seed waste and kernels has become economically viable due to the advent of an issue linked to a massive waste disposal problem of potentially valuable resources. This type of waste showed promising results in an industrial setting, and was utilized by its addition to certain materials, such as: food packaging, polyurethane foams and even batteries, as well as being used as potential feedstock to produce biofuels and activated carbon.

### 7.2. Apricot Seed Waste in Polymer Production

#### 7.2.1. Food Packaging

Kernels of bitter apricots—a major agricultural waste—were used to extract apricot kernel essential oil (AKEO). One important component of which is N-methyl-2-pyrrolidone (NMP), a powerful antioxidant, antimicrobial agent, present in a significant quantity.

AKEO was incorporated with chitosan with the intent of producing food packaging films. Chitosan is an abundant biopolymer that exhibits non-toxic, antimicrobial and biodegradable properties [61], the extracted kernel oil was added to examine the extent of which these films could be improved. The films were prepared with the solvent casting method, and the incorporation of AKEO was verified by Fourier Transform-Infra Red (FT-IR) spectra, as well as Field Emission Scanning Electron Microscope (FE-SEM) images. 

**Figure 6:**
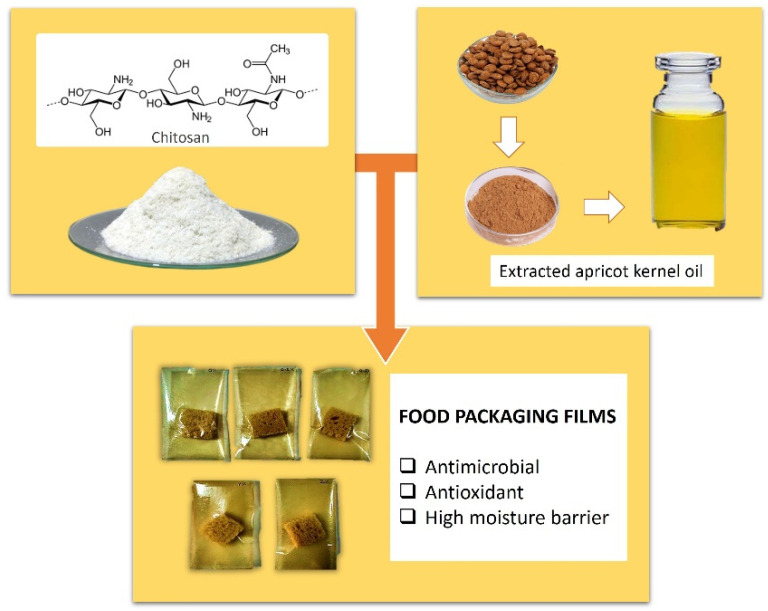
Chitosan films incorporated with AKEO [62].

The revised films demonstrated better water resistance and 41% improved water vapor barrier quality, when chitosan to AKEO ratio is 1:1. The elongation percentage value considerably increased, with oil ratio of 0.125 with respect to chitosan, however, a significant drop resulted with further addition. Despite that, a continuous increase in tensile strength value with increasing oil concentrations and a 94% improvement were noticed in the film with equal AEKO to chitosan ratio. antimicrobial and antioxidant properties were noticeably greater in films embedded with AKEO compared to regular ones made without its addition, as they were successful in the inhibition of fungal growth on packaged bread pieces (Figure 6) [62].

#### 7.2.2. Polyurethane Composites

**Figure 7:**
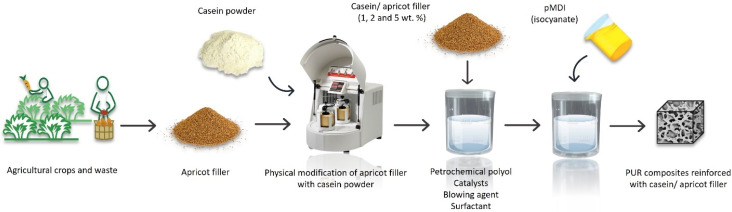
Making of PUR composites reinforced with apricot/casein filler [65].

Polyurethane (PUR) composites are broadly used in a variety of applications, which could be contributed to the various raw materials from which they could be acquired, such as: polyols and poly-isocyanates, with the addition of certain modifiers such as catalysts, fillers, flame retardants and more [63]. With the majority of the basic ingredients being acquired from non-renewable petrochemical raw materials. PUR foams are usually obtained using the one step method, two-component system; component A is a polyol system with the addition of the aforementioned modifiers, component B is an isocyanate system. Isocyanates are highly reactive molecules with low molecular weight, that are usually associated with health issues [64]. They are primarily used as fillers in PUR foams, acting similar to a binder. Moreover, polyols and isocyanates could be obtained from natural waste, and the fatty acid rich composition of the apricot stone allows it, thus a natural renewable source is accessible. The filler considered was a combination of apricot stones—a source of bio-polyols and isocyanates—and casein which is a natural flame-retardant; due to presence of substantial amounts of nitrogen and phosphorus, which use proved effective in previous studies. This particular mixture was considered to investigate the possibility of simultaneously improving the mechanical properties and the reduction of flammability. The filler was prepared with mechanically ground apricot stones, that later were sieved and mixed with casein powder with 1:2 ratio—by weight [65]. Additionally, the filler underwent a physical modification. It was used as the reinforcing filler in the making of PUR composites (rigid PUR foams) and was incorporated at different weight percentages (1, 2 and 5 wt.%), the other components were also added by weight (Figure 7).

It was observed that the addition of the apricot/casein filler affected the morphology of the PUR composites, as the cellular structure became less homogenous and the closed-cell content decreased, with increasing percentages of the filler. Thermal conductivity is affected by closed-cell content, as a decrease exhibited in closed-cell content leads to increased thermal conductivity, which is an important factor in thermal insulation materials [65]. The addition of the apricot/casein filler by 1 wt.% and 2 wt.% showed better results in comparison with 5 wt.%, 1 wt.% and 2 wt.% [65] demonstrated an improvement in mechanical strength (compressive strength, flexural strength, impact strength), while the 5 wt.% had significantly deteriorated [66]. Additionally, improved thermal stability as well as flame retardancy were observed (Table 4).

The best results observed were of the foams incorporated with 2 wt.% of apricot/casein filler, and that suggests that the modified filler could be used as low-cost, effective, environmentally friendly alternative.

### 7.3. Apricot Seed Waste in Biofuel and Activated Carbon Production 

Oil was extracted from apricot seed kernel, with a yield of 49.44% *w*/*w* of kernels. The selection of potassium hydroxide (KOH) could be contributed to its properties as it has low activation temperatures and produces higher yields [67]. KOH was used as the catalyst for the transesterification—displacement of alcohol from an ester with apricot seed kernel oil with methanol and ethanol to produce methyl and ethyl, respectively. The characteristics of the acquired biodiesels were evaluated and found to comply with the American Society for Testing and Materials (ASTM) D6751 limits. Pyrolysis of the de-oiled seed kernel occurred in a semi-batch reactor for bio-oil production. Various parameters affecting the yield of bio-oil were studied: pyrolysis temperatures, pyrolysis time, feed particles size. The greatest production of bio-oil (43.66% *w*/*w*) was accomplished at: pyrolysis temperature 450 °C, pyrolysis time 60 min, feed particles size 0.25 mm [68].

The bio-oil acquired under the optimal conditions was characterised by elemental analysis, FTIR spectroscopy, as well as column chromatography. The FTIR analysis of the biofuel showed that it is composed mostly of alkanes, alkenes, ketones, carboxylic acids, and amines. The characteristics of the bio-oil were analysed with reference to calorific value, density, flash point, pH, acid value, pour point, and refractive index [68,69]. The characteristics were similar to those of petroleum fractions and comparable to those of different bio-oils published in previous literature. The resulting bio-oil can be employed to be used in place of traditional, commonly used fuel and chemicals.

**Figure 8:**
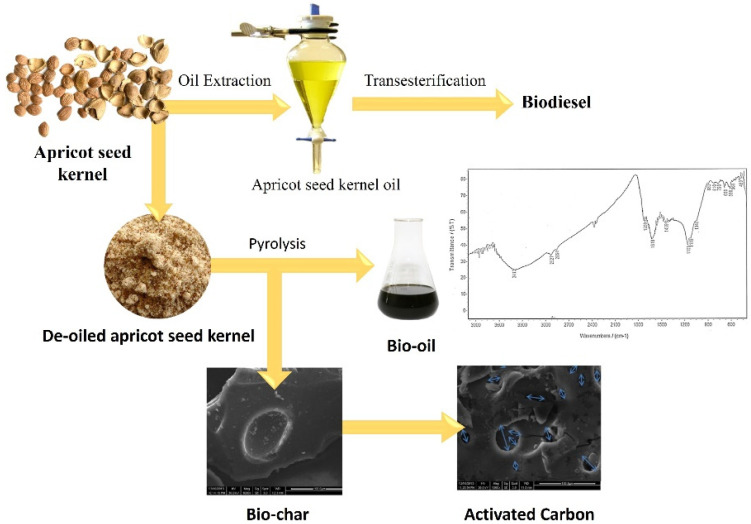
Production process of biofuel and activated carbon from apricot seed kernel [68].

The chemical activation method, using KOH as the activating agent was used to convert the biochar into activated carbon. The effect of the process parameters; the activation temperatures, activation time, and feed particles size on the yield, Iodine adsorption, as well as the surface area of the acquired activated carbon were studied. The better activated carbon sample was acquired at activation temperature 600 °C, activation time 90 min, feed size 60 mesh. The obtained activated carbon demonstrated good characteristics for the potential use in the removal of organic micro-pollutants from wastewater, as well as heavy metal adsorption, as demonstrated by scanning electron microscopy (SEM), as well as FTIR spectroscopy (Figure 8) [68,69,70].

### 7.4. Apricot Seed Waste in Batteries 

**Figure 9:**
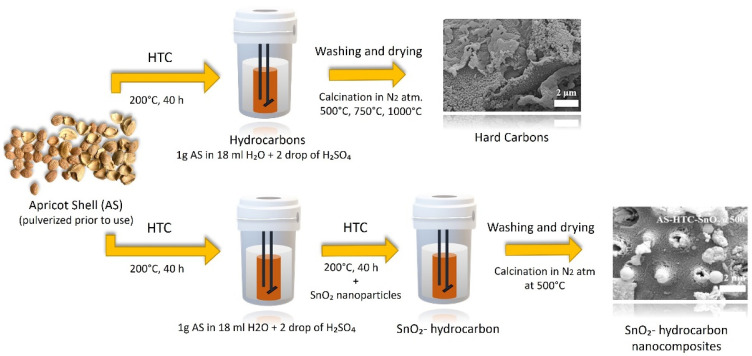
Production of anode materials for sodium ion batteries from apricot shell [71].

Apricot shell waste was employed in this application and was fed to sodium ion batteries. Hydro-thermal carbonization was used to create hard carbon anode materials from apricot shell waste (HTC) [71,72]. Additional pyrolysis at various temperatures was used to achieve acceptable conductivity and a larger surface area, both of which are important for battery applications. Their goal was to have a surplus of sodium storage capacity values. Under HTC circumstances, SnO_2_ nanoparticles are also added to the generated carbons obtained from apricot shell waste. Over 250 cycles, maximum capacity values were obtained using 1000 °C treated hard carbon anode materials with 184 mAh/g of capacity. The SnO_2_ hard carbon anode preparation method results in significantly improved electrochemical characteristics. Mechanically combining SnO_2_ with hard carbon, on the other hand, results in quick fading capacity (Figure 9) [71].

The use of agricultural waste such as seeds and kernels are a smart move, as they are frequently overlooked by-products despite their numerous characteristics. For example, they are non-edible and thus do not compete with the demand for food supply; they are also a renewable, cost-effective, and environmentally friendly source of energy. Rather than being disposed of, they could be repurposed to benefit humanity and alleviate some of the environmental burden. The use of such waste in food packaging is an excellent idea, as it comes from a renewable, biodegradable source that poses fewer environmental risks than conventional plastic. Additionally, its use as a filler in polyurethane materials would result in a more thermally stable and environmentally friendly product. Utilizing it as a feedstock to produce liquid biofuels may be a more accessible and cost-effective option. Incorporating apricot seed waste and utilizing it proved effective and with such promising results in each application, it would open doors for more in the future, and with that perhaps the environment would recover.

## 8. Conclusions and Future Perspectives

The focus of this review is to summarize the most recent research in this field with a focus on therapeutic action, phytochemicals, and industrial applications of apricots. In conclusion, apricots have many benefits in the medical field as it contains many antioxidants, anti-inflammatory, antidiabetic, hepatoprotective, antimicrobial and antiviral properties that could benefit the medical field. This is an updated review about the significance of apricot as the most widely produced fruit in the world, as well as the various applications for its waste in the food and functional food industries. There is a dearth of trustworthy published review data, estimating the use of apricots that promising future in its applications as an element in the industrial field, and the advantages that can be realized. We highlight the significance of apricot and its waste benefits in food packaging, biofuels, and batteries, which are areas that still need the scientific community’s attention. To further understand the mechanism underlying the apricot’s capacity to lower risk of various disease, researchers should continue to investigate the interactions between the numerous phytochemicals found in apricots and diseases. 

## Figures and Tables

**Table 1:** Nutritional values of apricot (fresh and dried fruits) [20].

Nutrient	Nutritional Values per 100 g		
	Raw Apricot	Dried Apricot	Kernel
Water	86.35 g	30.89 g	-
Sugars	9.24 g	53.44 g	17.5–35.6 g
Fibres	2.00 g	7.30 g	11.85–13.6 g
Proteins	1.40 g	3.39 g	14.6–27.1 g
Lipids	0.39 g	0.51 g	2.1–3 g
**Minerals**			
Calcium	13 mg	55 mg	0.0076 g
Iron	0.39 mg	2.66 mg	0.0042 g
Magnesium	10 mg	32 mg	0.003 g
Phosphorus	23 mg	71 mg	0.0028 g
Potassium	259 mg	1162 mg	-
Sodium	1 mg	10 mg	0.0011 g
Zinc	0.2 mg	0.39 mg	-
**Vitamins**			
Vitamin C	10 mg	1 mg	-
Thiamin	0.03 mg	0.015 mg	-
Riboflavin	0.04 mg	0.074 mg	-
Niacin	0.6 mg	2.589 mg	-
Vitamin B6	0.054 mg	0.143 mg	-
Vitamin E	0.89 mg	4.33 mg	0.003–0.040 g
Folate	9 µg	10 µg	-
Vitamin A	96 µg	180 µg	-
Vitamin K	3.3 µg	3.1 µg	-
Vitamin A (International Unit)	1926 IU	3604 IU	-

**Table 2:** The nutritional and chemical composition of some apricots.

	Varities	Hacıhloğlu	Hasanbey	Soğancı	Kabaası	Cöloğlu	Cataloğlu
Quality							
Total phenolics		5341.29 g	5827.98 g	4965.99 g	5822.03 g	5674.25 g	6107.21 g
β-carotene		21.87 g	22.02 g	9.18 g	26.18 g	5.74 g	17.53 g
Total Carotenoids		21.87 g	50.78 g	23.29 g	40.00 g	14.83 g	32.08 g
Sucrose		22.96 g	35.96 g	25.69 g	39.00 g	34.89 g	24.98 g
Glucose		19.21 g	14.72 g	17.16 g	18.64 g	18.95 g	21.40 g
Fructoses		13.56 g	12.16 g	13.99 g	13.05 g	15.68 g	15.02 g
Sorbitol		26.80 g	16.91 g	22.66 g	19.14 g	24.35 g	26.84 g
Citric Acid		776.2 g	739.7 g	725.7 g	923.1 g	431.8 g	436.5 g
Malic Acid		1814.8 g	2341.1 g	1092.4 g	1279.0 g	1312.9 g	973.4 g
Ascorbic Acid		37.7 g	49.3 g	28.5 g	41.6 g	20.6 g	27.9 g
Potassium		1849 g	1811 g	1879 g	1880 g	1227 g	1377 g
Calcium		102.3 g	100.7 g	110.0 g	105.7 g	87.0 g	140.8 g
Sodium		10.9 g	8.8 g	8.9 g	12.6 g	14.0 g	13.9 g
Phosphor		107.0 g	118.6 g	97.9 ± 4.5	97.0 g	72.0 g	88.9 g
Magnesium		134.7 g	152.2 g	110.4 g	131.0 g	120.4 g	131.7 g
Iron		2.98 g	2.80 g	3.48 g	2.34 g	3.73 g	2.73 g
Selenium		0.150 g	0.190 g	0.115 g	0.150 g	0.230 g	0.145 g
Zinc		1.38 g	1.41 g	1.90 g	2.63 g	1.61 g	2.19 g
Manganese		1.41 g	1.59 g	1.24 g	1.66 g	1.71 g	1.58 g
Nickel		0.325 g	0.440 g	0.325 g	0.430 g	0.390 g	0.350 g

**Table 3:** The nutritional and chemical composition of some apricots.

	Varities	Hacıkız	Tokaloğlu	Alyanak	Iğdır	Bursa
Quality						
Total phenolics		6592.38 g	4233.70 g	6773.43 g	5823.76 g	8180.49 g
β-carotene		13.05 g	21.59 g	48.69 g	13.44 g	42.18 g
Total Carotenoids		22.81 g	50.07 g	91.75 g	25.26 g	91.89 g
Sucrose		30.07 g	56.83 g	41.27 g	34.83 g	49.87 g
Glucose		23.67 g	11.38 g	18.33 g	17.06 g	9.47 g
Fructoses		11.03 g	7.77 g	6.53 g	11.95 g	6.34 g
Sorbitol		19.87 g	5.05 g	2.47 g	6.37 g	3.38 g
Citric Acid		443.9 g	2055.2 g	3524.5 g	7697.3 g	9997.1 g
Malic Acid		1347.8 g	2656.2 g	3888.9 g	3030.4 g	3930.0 g
Ascorbic Acid		37.1 g	45.8 g	38.3 g	68.4 g	96.8 g
Potassium		1605 g	1926 g	2319 g	3219 g	3455 g
Calcium		173.6 g	113.6 g	240.5 g	233.7 g	230.2 g
Sodium		15.9 g	8.0 g	10.1 g	17.8 g	11.7 g
Phosphor		104.6 g	144.1 g	157.2 g	237.9 g	177.6 g
Magnesium		146.7 g	148.8	160.4 g	222.0 g	284.4 g
Iron		3.51 g	5.09 g	7.74 g	7.94 g	11.3 g
Selenium		0.185 g	0.250 g	0.335 g	0.310 g	0.400 g
Zinc		2.02 g	2.05 g	2.54 g	4.24 g	3.38 g
Manganese		2.29 g	1.97 g	2.85 g	2.67 g	2.85 g
Nickel		0.645 g	0.510 g	0.715 g	0.425 g	0.575 g

**Table 4:** Value comparison of reference foam, 1, 2 and 5 wt.% [65].

	Reference Foam	1 wt.%	2 wt.%	5 wt.%
**Mechanical strength**				
Compressive strength (Measured parallel) (Measured perpendicular)		+~5% +~7%	+~10% +~9%	−~11% −~18%
Flexural strength		+~3%	+~6%	−~6%
Impact strength		+~2%	+~4%	−~6%
**Flammability**				
T_max_ (°C) at stages of thermal decomposition: 1st 2nd 3rd	205 °C 313 °C 587 °C	221 °C 315 °C 587 °C	219 °C 317 °C 589 °C	217 °C 313 °C 591 °C
Char residue (wt.%) at 600 °C	27.6	30.3	31.0	31.9

## Data Availability

Not applicable.
